# Effect of Mediterranean Dietary Pattern on Cognitive Status in Community-Dwelling Older Adults

**DOI:** 10.3390/nu15183911

**Published:** 2023-09-08

**Authors:** Nathalie Yaghi, Rita El Hayeck, Christa Boulos, Marianne Abifadel, César Yaghi

**Affiliations:** 1 Department of Nutrition & Dietetics, Faculty of Pharmacy, Saint Joseph University, Beirut 1107 2180, Lebanon; christa.boulos@gmail.com; 2 Department of Geriatrics, Working Group on Dementia at Saint Joseph University (GTD-USJ), Faculty of Medicine, Saint Joseph University, Beirut 1107 2180, Lebanon; rihayeck@gmail.com; 3 Laboratory of Biochemistry and Molecular Therapeutics, Faculty of Pharmacy, Pôle Technologie-Santé, Saint Joseph University, Beirut 1004 2020, Lebanon; 4 Department of Gastroenterology, Faculty of Medicine, Saint Joseph University, Beirut 1107 2020, Lebanon; cesar.yaghi@usj.edu.lb; 5 Hôtel-Dieu de France of Beirut University Hospital, Beirut P.O. Box 166830, Lebanon

**Keywords:** dietary pattern, older adults, aging, cognitive impairment, Mediterranean diet, Westernized diet, Mediterranean country, cognitive decline

## Abstract

Modifiable factors associated with cognitive decline (CD) require more attention, particularly dietary patterns. This study aimed to investigate the link between cognitive decline and associated factors, particularly dietary patterns (DPs), in community-dwelling older Lebanese of modest economic status. Our cross-sectional national study included 352 participants above 60 years old, from the medico-social centers of the ministry of social affairs all over the country. CD was screened based on literacy. Nutritional and dietary data were collected through a validated food frequency questionnaire. DPs were extracted by the K-mean cluster analysis. CD was found in 32.7% and 61.5% of literate and illiterate groups, respectively. Identified DPs included a Westernized type and Mediterranean type, with high and moderate food intakes. In the context of literacy, independent factors associated with CD were age above 80 years, living in Beirut, frailty, and adopting a Westernized (OR = 3.08, 95% CI: 1.22–7.8) and a high-intake Mediterranean DP (OR = 2.11, 95% CI: 1.05–4.22). In the context of illiteracy, the same factors were associated with CD, but not DP nor frailty, with an age cut-off at 78 years. In a Lebanese sample of older adults, factors associated with CD depend on the level of literacy, with DP only associated with CD in the context of literacy.

## 1. Introduction

Life expectancy is increasing in all countries around the world, leading to a rise in the proportion and number of older people. According to the United Nations, the proportion of people over age 65 is expected to be 16% by 2050, compared to 9% in 2019. This increase will accelerate in the coming decades, particularly in developing countries [1,2,3].

With this global trend, the search for successful aging has become an important matter to societies, especially with the rise of age-related diseases. Cognitive impairment (CI) is considered one of the most common geriatric syndromes. Its presence poses major risks to the older population including greater disability, reduced quality of life, and higher morbimortality [4].

The brain undergoes neural development until approximately the age of 30 years, after which a slow process of atrophy takes place, with clinical signs of neurodegeneration occurring at an older age [5]. Normal CD occurs at a certain pace, and some factors, whether pathological or non-pathological, affect the progression of this decline, leading to a more rapid deterioration of functions, CI, and earlier onset of dementia. The trajectory of CD to dementia is affected by the interaction of genetic, endogenous, and environmental factors [6]. Rapid CD has often been linked with frailty, and the role of nutrition in CD has also been retained by researchers as a modifiable risk factor [4,6,7,8,9,10]. Healthy lifestyles may decrease the rate of CD and improve neuroplasticity. Modifiable factors include physical activity, healthy diet, mental stimulation, avoiding excessive exposure to neurotoxins (e.g., alcohol), treating depression and managing stress, and controlling common medical conditions such as hypertension, diabetes, and obstructive sleep apnea [11].

The Lancet Commission estimates that approximately 40% of dementia cases are attributable to a combination of 12 potentially modifiable risk factors, among which are low educational level (LEL), midlife hypertension and obesity, late-life depression (with bidirectional association), hearing loss, diabetes, physical inactivity, social isolation, smoking, and diet [12]. It has been reported that skeletal muscles, by secreting neurotrophic factors, affect brain function and its motor units [13].

Specific nutrients and phytonutrients in food have been found to have a protective role in brain aging [14,15,16,17,18,19]. Indeed, low nutritional status of vitamins (B6, B9, B12) have been found to be associated with higher rates of dementia, cardiovascular disease, and stroke [15,20]. The use of vitamin K antagonists has been associated with CI and lower gray matter volume in the hippocampus in geriatric patients [16]. The antioxidant vitamins C and E, the amino acids tyrosine and tryptophan, necessary for the synthesis of dopamine and serotonin, are also involved [21], as well as long-chain ω-3 fatty acids, which are essential components of neuronal membranes [22,23,24,25,26,27,28]. Existing evidence suggests that food ingredients, in particular specific phytonutrients found in berries, coffee, tea, olive oil, and ω-3 fatty acid-rich foods, have the potential to beneficially affect cognitive function. On the other hand, trans fats and saturated fats have a detrimental effect [28,29,30,31,32,33,34].

The most promising studies focus on the link between types of diets rather than specific nutrients and the risk of CD [14,35,36]. This whole-diet approach has been described as the most optimal preventive approach and is based on the synergistic interaction between several nutrients, as suggested by findings from the Three City observational study [22]. The Mediterranean diet and its correlates, like the MIND diet, characterized by a high intake of whole cereals and legumes, fruits, vegetables, and fish, are among the best described diets, with shown protective effects among several populations [36,37,38,39,40,41,42].

Healthy diet alone or diet combined with physical activity and cognitive training are also considered beneficial cumulative lifestyle modifications that have promising results in the prevention of CD; they have the potential to improve cognitive performance and delay neurodegeneration [43].

Among countries in the Arab world, Lebanon reported in 2015 one of the highest proportion of people aged 65 years and above, and this proportion is expected to become, by 2030 and 2050, 14.0 and 23.3%, respectively [44]. In a cultural context where the family is the primary provider of both financial and social care, the age dependency ratio is expected to shift in the near future from providing for children to supporting older people [44,45,46,47,48]. With more than 50% of older adults already considered in 2015 as economically deprived, decades of political and economic turmoil, aggravated by a sharp financial decline since October 2019, the Beirut port blast, and the Covid-19 pandemic, resulted in further limiting resources for older people [44,49] and making this age group rank higher in terms of priority of healthcare for the most vulnerable.

In Lebanon, the age-standardized prevalence of dementia is 9% in individuals above 65 years of age. Such figures make dementia prevalence in this country rank high within the global range of estimates [50]. Studies exploring associated factors of CD in Lebanon are scarce [50,51,52,53] and few investigated its association with nutrition-related parameters [54]. Determining the impact of risk factors and diet on CD is therefore of high importance.

The causal link between CD and diet remains to this day inconclusive, hence the interest of this trial, given that Lebanon is one of the countries where the Mediterranean diet is part of the food heritage [14,55].

The main goals of this study are therefore to explore the association of nutritional, dietary, and other modifiable factors on CD as well as evaluate the dietary habits and nutritional intake of a national sample of older Lebanese and estimate the prevalence of CD among this age category, contributing to a better understanding of this health issue.

## 2. Materials and Methods

### 2.1. Study Design and Participants

The national, cross-sectional study was carried out from October 2017 to October 2019, in seven of the eight governorates of Lebanon, in collaboration with the Ministry of Social Affairs (MOSA) through 77 medico-social centers serving low- to middle-income families. A sample of 600 individuals was initially targeted, including individuals aged 60 years and above who were community-dwelling and attending the MOSA socio-medical centers for medical care and social assistance. Data were finally retained for 401 participants, of whom 352 (88%) had a valid Food Frequency Questionnaire (FFQ). Details of the study, recruitment of participants, and data collection are described elsewhere [56].

### 2.2. Cognitive Status Screening Tools

Screening and diagnosis of cognitive impairment were conducted using 2 instruments, the Adapted Arabic versions of Mini-Mental State Examination (A-MMSE) and Test des Neufs Images (A-TNI-93) [57,58]; these tools were administered to the literate and illiterate participants, respectively.

The MMSE is one of the most frequently used screening instruments for dementia. The MMSE is an 11-item assessment tool used to evaluate global cognitive performance. It has well-documented reliability and validity. Trained investigators administered the MMSE to participants, measuring 5 domains: orientation, memory, attention, verbal skills, and visuospatial reasoning for a maximum score of 30 points. It is easy to administer and takes 5 to 10 min. In our study, we used the Lebanese population-specific cut-offs for the MMSE suggested by El Hayeck et al., based on gender, age, and educational level [57].

The TNI93 is a test designed to evaluate episodic memory in illiterate population samples. Subjects are asked to learn images using the principle of encoding specificity. The TNI93 does not require the use of written language or the ability to read and can therefore be administered in any language. The duration of the test is around 10–15 min. Participants having scored at or below the population-specific cut-offs, below or equal to 6 for the TNI Free Recall or a TNI Total Recall score below or equal to 8, were considered as having low cognitive status [58,59]. Since, to our knowledge, no previous research showed interchangeable use between MMSE and TNI, and with education being an important component of cognitive reserve preservation and determinant of cognitive decline rates in elderly [50,60], participants were split into 2 groups for analysis, and data concerning CD were dichotomized based on literacy and analyzed separately.

### 2.3. Sociodemographic-, Health-, and Nutrition-Related Data

The 7 governorates of Lebanon were divided into 5 regions: Beirut and suburbs, Mount Lebanon, North, South, and Bekaa regions.

Literacy was evaluated based on the capacity of participants to read or write. Educational level was classified into several categories based on the obtainment of official degrees: elementary, complementary, secondary, and university diplomas. Depressive tendency was evaluated using the Arabic version of the Geriatric depression scale, the A-GDS5 [61,62]. Use of anti-depressant and anti-anxiolytic medications was reported. Data were then transformed into 2 categories. The first category included people with a tendency for depression; they either had a score of GDS5 ≥ 2 or were taking medication to treat anxiety and/or depression. The second category included people with GDS5 < 2 who were not taking any medication to treat depression or anxiety.

Other data collected are described in a previous publication [56].

Nutritional status was estimated using the Mini-Nutritional Assessment Short Form (MNA-SF) [63,64], classifying individuals into three levels: normal nutritional status (score above 12), at risk of malnutrition (scores 8–11), and malnutrition (scores below 8). Participants were then classified into two categories. The first category, called “poor nutritional status”, included participants that were considered malnourished and at risk of malnutrition by the MNA-SF, and the second category, called “normal nutritional status”, included participants who had a normal nutritional status according to MNA-SF.

Nutrient intake and dietary patterns were extracted from the validated FFQ [64]. Calculation of the Nutrient Adequacy ratio (NAR) of 17 selected nutrients was computed. Nutrients included vitamins A, D, E, K, C, B1, B2, B3, B6, B9, B12, and minerals such as calcium, phosphorus, magnesium, iron, selenium, and zinc. The NAR was calculated by dividing the estimated nutrient intake of individuals by the age and sex-specific recommended dietary allowance (RDA) for these nutrients, according to the established dietary reference intake (DRI) recommendations [64,65]. For all nutrients, RDA values were used, except for vitamin K, where adequate intake (AI) was used as the DRI value for comparison.

To extract dietary patterns, the 90 foods listed in the FFQ were then grouped into 20 predefined categories based on similarities in nutrient composition and consumption characteristics. After running the cluster analysis, three dietary patterns were identified in the total sample. The first, called the Westernized-type dietary pattern (WDP), was characterized by the highest caloric intake, consumption of refined flour products, sugar and sweets, dairy products, as well as processed and saturated fats and the lowest olive, seed, and oleaginous fruit and whole cereal product intake. The second pattern, called the high intake/Mediterranean-type dietary pattern (HIMEDDP), was characterized by a relatively high caloric intake, a higher consumption of vegetables, fruits, legumes, than the other 2 DPs, and the highest consumption of foods rich in monounsaturated fats. Median consumption of olive, seeds, and oleaginous fruits in the HI-MEDDP group was above nine teaspoons of oil equivalent per day. This pattern also had the lowest consumption of refined flour products, and the highest consumption of whole cereal products. Finally, the third pattern, called the moderate intake/Mediterranean-type dietary pattern (MOD-MEDDP), was characterized by a diversified and balanced DP. Consumption of most foods in this pattern was either intermediate or lower compared to the other two patterns, with the lowest consumption of sweets and sugar among the three patterns [56].

### 2.4. Statistical Analysis

Statistical analysis was performed using the SPSS program version 21.0. For numerical variables, data were expressed as mean ± standard deviation and median (interquartile ranges), and as numbers and percentages, for categorical variables.

Differences between cognitive status characteristics for sociodemographic, nutritional, dietary, health-related, and anthropometric data were compared using the non-parametric tests, Mann–Whitney and Kruskal–Wallis for numeric variables that are not normally distributed, and chi-square test for categorical variables. K-means clustering was used to regroup participants with similar dietary patterns. Differences in food intake between dietary patterns were evaluated by Kruskal–Wallis test.

Cut-offs for age, polypharmacy, multimorbidity, age-related conditions, and waist-to-height ratio (WTHR) were determined through a classification tree and used for subsequent multivariate analysis. Age cut-offs at 80 years and 78 years were determined, respectively, for the literate and illiterate groups for a specificity of >80%. In the multivariate analyses, we proceeded to several models of binary logistic regression using the dichotomized CS variable as the dependent variable. Odds ratios (ORs) with 95% confidence intervals were calculated. The main explanatory variables reaching significance level identified in each model were added to the following model. In the multivariate analysis, age above 80 years and 78 years for the literate and illiterate groups, respectively, level of education (more than 13 years) (only for the literate group), female gender, living conditions, marital status, income, and living in Beirut were entered first (model 1). From this model, we retained covariates significantly associated with CD and then added anthropometric and health-related variables, including multimorbidity, polypharmacy, frailty, presence of age-related conditions, health perception, poor mental health, BMI categories, WTHR above 0.718, malnutrition, and level of physical activity (model 2). From model 2, we retained all covariates significantly associated with CD and then added the 3 dietary patterns (model 3).

The level of significance was fixed at *p* = 0.05, unless otherwise noted, for all analyses.

## 3. Results

### 3.1. Description of the Total Sample

Characteristics of the total sample population were published elsewhere [56].

Three types of dietary patterns were identified in the total sample: a Westernized-type dietary pattern (WDP), adopted by 11.9% of the participants, a high intake/Mediterranean-type dietary pattern (HI-MEDDP), followed by 23% of participants, and a third pattern, called the moderate intake/Mediterranean-type dietary pattern (MOD-MEDDP), representing the highest proportion of our sample (65.1%). Details of DPs are published elsewhere [56].

### 3.2. Cognitive Status and Characteristics of the Sample Based on Literacy

The total number of participants scoring low on either cognitive test (MMSE and TNI) was 145 (41.2%). The illiterate group accounted for 29.5% of the sample population and the literate group for 70.5%. In the total sample, the group with low cognitive results, compared to the one with good cognitive results, had more than double the proportion of people aged 80 and above, with 29% and 12.1% respectively, a higher proportion of dependency (24.1% vs. 10.2%), malnourishment (9.7% vs. 2.4%), and frailty (23.4% vs. 9.2%), and a higher proportion of participants with more than one age-related condition (60.7% vs. 48.8%), and a lower proportion of good health perception (31.3% vs. 50.2%). No gender difference was observed in the prevalence of CI (Supplementary Table S1).

The characteristics of literate and illiterate participants reflect almost the same characteristics as the total sample. The characteristics of the participants according to their cognitive test results are presented based on literacy level in Table 1 and Table 2.

Low CS was found in 32.7% and 61.5% of the literate and illiterate groups, respectively.

When only participants with low CS were compared based on literacy (Supplementary Table S2), illiterate participants with CI were older, with a mean age of 76.5 and 72 years, had a higher proportion of women (62.5% vs. 40.7% *p* = 0.009) and of participants considering their income insufficient income (70.3% vs. 53.1% *p* = 0.035), a higher proportion of high WHTR (>0.718) (34.9% vs. 10.7%), and poorer mental status (71.55 vs. 55% *p* = 0.038). The illiterate group with CI also had a higher proportion having more than one disease, age-related conditions, and poor self-rated health.

**Figure 1:**
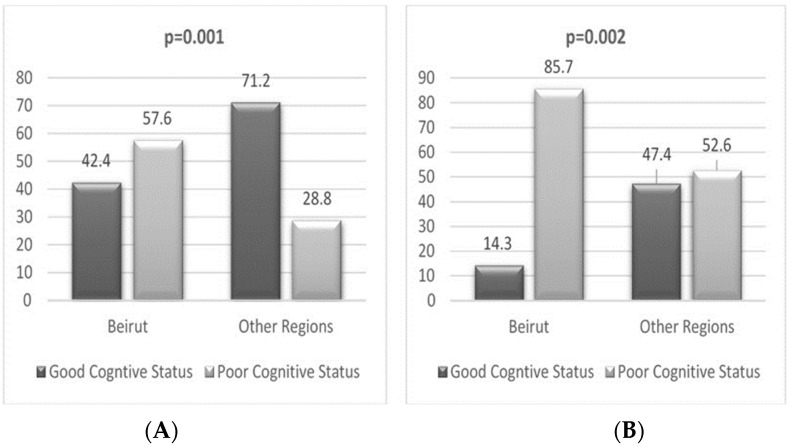
Regional distribution of participants based on cognitive status for the literate (**A**) and illiterate (**B**) groups.

The characteristics of participants living in the capital vs. the rest of the regions are found in Supplementary Tables S3–S6. The regional distribution of cognitive status is shown in Figure 1.

### 3.3. Cognitive Status and Nutrient Adequacy

In the total sample as well as in the literate and illiterate groups analyzed separately, NARs were found to be inadequate for Ca, vit D, and ω3-FA, for both levels of CS. No difference was found in the prevalence of inadequacy between the two levels of CS except for vitamin K and fiber in the group of illiterate, where the group with low CS had a significantly higher proportion of inadequacy than the group with good CS, with 19% vs. 2.5% and 51.6% vs. 27.5%, respectively, for vitamin K and fiber intakes, respectively.

### 3.4. Cognitive Status and Food Intake

In Supplementary Table S7, when comparing the two levels of CS, in the literate group, results were similar to those described in the total sample regarding the food intakes, showing a significant difference in low-fat sweet intake and sugars and jam portions. In the illiterate group, no difference was found in food intake between participants with low and good CS.

### 3.5. Dietary Patterns and Associated Factors

The multivariate logistic regression analysis performed in the total sample included 352 individuals with a complete set of data each and fulfilling all criteria of inclusion (Table 3). In the first model, the only socio-demographic factors associated with low cognitive status (LCS) included age above 80 years and 78 years, for the literate and illiterate participants, respectively, and living in Beirut. In the second model, in addition to previous factors, anthropometric and health-related factors associated with CD were frailty and bad health perception. In the final model, by adding dietary patterns, independent factors associated with CD were advanced age, living in Beirut, bad health perception, and adopting a HI-MEDDP as compared to MOD-MEDDP pattern.

The same regression analysis was performed in the literate and illiterate sub-samples including 248 and 104 participants, respectively (Table 4 and Table 5).

Within the literate sample (Table 4), in the first model, factors associated with low cognitive status (LCS) included age above 80 years and living in Beirut. In the second model, the only anthropometric and health-related parameters that were found to be associated with low cognitive status were frailty and BMI above 32 kg/m^2^. In the final model, by adding dietary patterns, independent factors associated with CD were age above 80 years, living in Beirut, frailty, as well as adopting WDP and HI-MEDDP patterns, as compared to the MOD-MEDDP pattern.

In the illiterate group, the same binary regression analysis performed (Table 5) showed in the first model that factors associated with LCS included age above 78 years, living in Beirut, and living conditions. In the second model, no anthropometric nor health-related parameters were found to be associated with LCS. In the final model, by adding dietary patterns, the factors associated with CD remained: above 78 years, living in Beirut, and living alone as compared to living with partners, with no association with dietary patterns adopted.

## 4. Discussion

This study showed no gender difference regarding cognitive impairment. In the total sample, factors associated with low cognitive test results were advanced age, living in Beirut, living alone compared with living with a partner, and bad health perception. Only adopting a HI-MEDDP was found associated with LCS. In the literate group, in addition to being 80 years old and above and living in Beirut, being frail and following a WDP and HI-MEDDP were also found to be independent factors associated with CD. In the illiterate group, identified independent factors associated with CD were being 78 years old and above, living in the capital, and living alone compared to living with partners.

This study suggests that age remains the strongest factor related to CD. Our results pointed out that age, above 80 years for the literate and 78 years for the illiterate, remains one of the most important non-modifiable risk factors related to CD, as already previously established [11,66].

The study findings did not show any difference in prevalence of CD between gender. With a higher proportion of illiteracy in women, the TNI test used in this study for illiterate participants only measures periodic memory and does not evaluate other domains of cognition like the MMSE, possibly underestimating CD in this category of participants [59,66]. According to the literature, female gender is another prominent risk factor described to be related to CD. Most studies reported that women are more prone to CD than men, mostly because they live longer and have lower educational and social attainment levels [12,50]. Furthermore, a meta-analysis pinpointed that reporting gender difference in the prevalence of CD must be taken with caution, and to better understand such a difference, especially in the early stages, a better knowledge of the type of CI should be made [57,67].

Differences existed between the literate and illiterate groups, suggesting that in the context of low cognitive reserve, risk factors might be different. Having a higher cognitive reserve, as observed previously with higher educational attainments, could be considered a protective factor for cognitive status and slow the age-related cognitive decline observed with aging, by possibly delaying the decline by 2 years [11,66]. Furthermore, DP does not impact CS like social status and social interactions, and particularly age. The latter overrides the impact of other factors, particularly DP [57,68,69]; in the illiterate group, DPs were not found to be associated with CD. In the literate group, although educational level did not stand as an independent associated factor with CD, some modifiable factors like frailty and dietary patterns were found to be associated with CD. This result could imply that in the context of literacy, i.e., with a higher cognitive reserve, possible modifiable factors such as frailty and diet can be worked upon to possibly change the course of CD. Nevertheless, further research should be tailored to better understand the role of education as a mediating factor for these modifiable factors and clarify the role of education, using a unified detection tool and different study design. Previous data in Lebanon, describing the importance of education and occupational attainment on cognitive reserve, suggested that reaching higher levels of education, engaging in complex occupations, and participating in more cognitively stimulating leisure activities enhance the cognitive reserve and allow older adults who attained high educational levels to be 7.1 times more likely to have better global cognitive function than others who attained lower education [50]. Cognitive reserve, characterized by neuro-elasticity, is the result of lifetime intellectual activities and environmental factors, allowing individuals with high cognitive reserve levels to better cope with the same amount of brain damage as opposed to individuals with lower levels [69]. Living alone compared to living with partners showed to be associated with CD in the illiterate group. Previous findings pointed out the importance of social interaction on the prevalence of CI [70,71].

Living in Beirut was one of the identified independent factors associated with CD in both groups of literacy. In contrast to most studies describing the association of urban vs. rural living in relation to CD, our study showed that living in Beirut increased the odds of CD by 5-fold. Participants living in Beirut were found to have the highest prevalence of multi-morbidity, age-related conditions, malnutrition, and insufficient income. They also had the highest proportion of inadequate nutrient intakes, also previously reported to be related to CD, like vitamin A, E, B9, Mg, and Ca. These parameters could be considered as confounding factors when comparing rural vs. urban prevalence of CD, since each of these factors is described by the literature as a risk factor for CD [7,72,73,74,75,76,77,78]. Some previous findings showed that rates of dementia among urban dwellers tended to be lower than those of rural dwellers. Theories explaining this difference focus on better access to higher-quality education, as well as public and health services among urban dwellers [51,70,79], but contextualization of the findings to Lebanon where older adults lack health coverage and retirement plans [48] would allow a better understanding of results. These findings are an opportunity to highlight the importance of directing the attention of healthcare not only to rural areas but also to urban areas with socio-economic difficulties.

Our study failed to find an association between nutrient intake and CD. Previous reports have documented the role of specific vitamins and nutrients as having a protective effect on CD. Vitamins B6, B9, B12, Mg, vitamin D, and W3-FA are among the studied nutrients [78,80,81,82].

Our study results pinpointed the difference in the prevalence of inadequate intakes of vitamins in the illiterate group with CI compared to the group with GCS, showing a possible role of sub-optimal intake of vitamin K in the development of CI. Vitamin K has been previously shown to be involved in the synthesis of sphingolipids, a major constituent of the myelin sheath, and in neuronal survival thanks to vitamin K-dependent proteins (VKDP proteins). The use of vitamin K antagonists has been associated with CI and lower gray matter volume in the hippocampus in geriatric patients [16].

Regarding food intake, in the literate group, in addition to intakes of low-fat sweets, a significant difference was observed with intakes of sugars and jams, as absolute quantity or relative per 1000 Cal. These findings were confirmed by the association of the WDP characterized by higher intakes of sugars. Mitigated results regarding the impact of sugar-sweetened foods and lower CS were reported in the literature. In a study using data from NHANES, high consumption of added sugar, above the 75th percentile of the sample population intake, was found to be associated with low CS (OR = 1.509, 95% CI: 1.109–2.052) [83]. Another study linked intake of total sugar, sugar-sweetened beverages, but not sugar-sweetened foods, to higher cognitive decline [84]. In the context of high BMI and high WTHR ranges, a high intake of sugar could be implicated in the physiopathology of development of CD, especially given the fact that sugar intake is described to be related to metabolic syndrome and cardiovascular diseases, described to have an association with CD [72]. Our study has paved the way to defining a possible threshold for sugar intake in relation to CD, making added sugar intake a new target for future studies as an associated factor for CD in a Mediterranean context.

Our main findings in the literate group reported that the adoption of a WDP is associated with 3-fold the odds of CD and a HI-MEDDP is associated with almost 2-fold the odds of CD, when compared to MOD-MEDDP. Some specific food components and specific nutrients were previously implicated and affect the course of development of CD. The underlying mechanisms by which they attenuate CI can be attributed to modulation of gut microbiota, to antioxidant and anti-inflammatory components, as well as the synergistic effect of food components in these DPs [35,38,41,85]. Several food combinations in specific dietary patterns have been studied in the context of cognitive decline, from MCI to dementia, in various settings, with some exhibiting favorable results and others showing no evidence of such effects. Previous studies associated specific dietary patterns like the Mediterranean diet (MedDiet) [37,86,87], dietary approaches to stop hypertension (DASH) diet [41], Mediterranean-DASH diet intervention for neurological delay (MIND), with the prevention of CD [35,41]. Among the most-studied DP is the Mediterranean diet and its correlates such as the MIND diet, the DASH diet, and the Healthy diet [19,34,36,42,88,89]. In the 3 C- city cohort, Bordeaux chapter, over 5 years follow up, higher adherence to the Mediterranean diet was associated with slightly slower MMSE decline but not with other measures of cognitive decline. The Mediterranean diet in the presence of other contributing factors is a better protector against decline than Med diet DP alone [36]. A major constituent of the Mediterranean diet is olive oil. Components in the olive oil, such as oleocanthal, have an anti-inflammatory effect, considered to be part of the beneficial effect of the Med diet, and other pro-inflammatory components, such as found in a Westernized-type DP, are considered to negatively affect CS [88,90,91,92,93].

Finally, a major factor associated with CD was frailty in the literate group, with frailty increasing the odds of CD by 4-fold. Whether CD causes increased frailty or frailty increases the risk of CD is not yet clearly understood in the literature. In the context of literacy, our findings suggest an association between frailty and CD, whereas in our analysis on frailty, educational level was not taken into consideration. Older adults with both physical frailty and CI are shown to be at higher risk of adverse health outcomes, including death, disability, hospitalization, and dementia, than those with either condition alone. Such findings could pave the way to the concept of cognitive frailty described in the literature and defined according to specific diagnostic criteria [4,6,94,95]. While the underlying mechanisms of cognitive frailty are still unclear, some factors were previously shown to be associated with cognitive frailty, such as sociodemographic factors, nutritional status, physical and cognitive activities, functional status, comorbidities, medication use, and gut-derived metabolites [10,95,96]. Further studies, taking into consideration the social determinants of health while focusing on the role of DP in the course of cognitive frailty, are required to clarify the mechanisms through which physical frailty and CI would interact particularly in the context of socio-economic insecurity in Lebanon.

### Limitations

We noted some limitations to this study. Selection and reporting bias might be limiting factors in reporting diseases. Low intake of some nutrients and vitamins in the total sample, such as vitamin D and W3, hindered the detection of impact of potential nutritional factors in relation to CD. Furthermore, lack of consistency among participants in reporting intakes of vitamins and minerals supplements caused the exclusion of this source of nutrients in the intake analysis, which might have an impact on outcome. Having a small number of participants adopting the WDP-DP could have also been a possible bias. Larger prospective studies are therefore required to further investigate the impact of dietary patterns on risk of CD with a special emphasis on sugar intake and BMI ranges, with equal distribution among DPs. Unifying screening tools in the detection of CI would also allow for more comparable results in relation to CI and help better define the role of education with this regard. A possible way of evaluating cognitive performance would be through clinical dementia rating (CDR) as a more precise tool to evaluate CI. The Washington University Clinical Dementia Rating (CDR) is a global scale frequently used in clinical and research settings to describe stage-dependent features of dementia and in clinical drug trials for measuring clinically meaningful changes. The clinical protocol incorporates interviews to obtain information necessary to rate the subject’s cognitive performance in six domains: memory, orientation, judgment and problem solving, community affairs, home and hobbies, and personal care [60].

## 5. Conclusions

In summary, our study was the first to explore the association between CD and specific dietary patterns extracted in a posteriori method, in adults over 60 years of age in Lebanon, using specifically validated questionnaires. Despite the difficulties in addressing this specific age group, we succeeded in showing that age, frailty, and adopting a Westernized-type DP, and high sugar intake DP remain the main associated factors related to CD in literate low-socioeconomic settings. Although our results did not evaluate CD through clinical dementia rating (CDR), they support the newly developed concept of cognitive frailty, defined as the presence of frailty concomitantly with cognitive decline (evaluated by the CDR). Most importantly, we demonstrated that in a literate group, adopting a WDP pattern with high sugar consumption and a HI-MEDDP were also linked to CD, and that a more moderate Mediterranean-like pattern was protective. Results also pinpointed a possible mediating effect of education on people’s susceptibility to risk factors, possibly overriding the effect of diet on the progress of CD. Illiteracy favors other factors such as living conditions, but future research would need to explore this. Results directed the attention to individuals living in the capital as being more vulnerable than those living in the rural areas and suburbs. Multi-domain preventive recommendations on a national level would need to tackle social determinants of health, particularly in a low socio-economic context, whether in the urban or rural areas, in addition to tackling prevention of age-related non-communicable diseases, in which diet is a pillar in prevention.

## Figures and Tables

**Table 1:** Socio-demographic characteristics of participants based on cognitive status, for the literate and illiterate groups.

		Literate 248 (70.5)			Illiterate 104 (29.5)		
		GCS 167 (67.3)	CI 81 (32.7)	*p*-Value	GCS 40 (38.5)	CI 64 (61.5)	*p*-Value
Age (y)		72 ± 7 (72)	73 ± 9 (72)	0.414	73.8 ± 6.4 (74)	77 ± 7 (77)	0.038
	≥80	21 (12.6)	22 (27.2)	0.004	4 (10)	20 (31.2)	0.012
Gender	Men	90 (53.9)	48 (59.3)	0.425	14 (35)	24 (37.5)	0.797
Regions	Beirut	14 (42.4)	19 (57.6)	0.001	4 (14.3)	24 (85.7)	0.002
	Other regions	153 (71.2)	62 (28.8)		36 (47.4)	40 (52.6)	
Education	Less than elementary	36 (21.6)	6 (7.4)	0.018			
	Elementary	44 (26.3)	36 (44.4)				
	Complementary	32 (19.2)	14 (17.3)				
	Baccalaureate	25 (15)	15 (18.5)				
	higher education	28 (16.8)	9 (11.1)				
Income	Insufficient	84 (50.3)	43 (53.1)	0.68	26 (65)	45 (70.3)	0.571
Marital status	Married	117 (70.1)	56 (69.1)	0.822	19 (47.5)	40 (62.5)	0.372
	Divorced	5 (3)	1 (1.2)		1 (2.5)	3 (4.7)	
	Single	10 (6)	6 (7.4)		1 (2.5)	1 (1.6)	
	Widowed	35 (21)	18 (22.2)		19 (47.5)	20 (31.2)	

Abbreviations: Y: years, GCS: good cognitive status, CI: cognitive impairment. Values are presented as mean ± standard deviation (median). *p*-value represent statistical significance for T-test or Mann–Whitney for, respectively, normal or non-normally distributed data. Categorical variables are represented as total count (percentage). *p*-value represents statistical significance for Pearson Chi-square.

**Table 2:** Health characteristics of participants based on cognitive status, for the literate and illiterate groups.

		Literate 248 (70.5)			Illiterate 104 (29.5)		
		GCS 167 (67.3)	CI 81 (32.7)	*p*-Value	GCS 40 (38.5)	CI 64 (61.5)	*p*-Value
Health and Functional Parameters							
HGS (kg)		26.2 ± 9.3 (25.6)	26.4 ± 9.1 (23.9)	0.874	22.3 ± 9 (19.7)	18.4 ± 7.6 (17.9)	0.026
	Low HGS	68 (43.3)	33 (45.2)	0.788	21 (52.5)	49 (77.8)	0.007
Physical Activity	Sedentary	132 (79)	58 (71.6)	0.3	33 (82.5)	52 (81.3)	0.91
	Regular active	34 (20.4)	23 (28.4)		6 (15)	11 (17.2)	
	Optimal active	1 (0.6)	0 (0)		1 (2.5)	1 (1.6)	
Poor mental health		70 (41.9)	44 (55)	0.054	23 (65)	46 (71.9)	0.460
Multi-morbidity	>1 disease	120 (72.3)	59 (72.8)	0.928	35 (87.5)	56 (87.5)	1
Age-related conditions	>1 condition	73 (43.7)	39 (48.1)	0.51	28 (70)	49 (76.6)	0.458
Polypharmacy		41 (24.7)	16 (20.5)	0.471	25 (62.5)	42 (70)	0.435
Frailty		10 (6)	15 (18.5)	0.002	9 (22.5)	19 (29.7)	0.421
Smoker	never	82 (49.1)	40 (49.4)	0.999	13 (32.5)	31 (48.4)	0.237
	present	52 (31.1)	25 (30.9)		19 (47.5)	21 (32.8)	
	previous	33 (19.8)	16 (19.8)		8 (20)	12 (18.8)	
Family history of CD		33 (19.8)	14 (17.3)	0.641	10 (19.6)	12 (22.6)	0.448
Nutritional status parameters							
Nutritional status	Malnutrition	3 (1.8)	8 (9.9)	0.004	2 (5)	6 (9.4)	0.415
BMI (kg/m^2^)		29.4 ± 5.6 (28.8)	27.9 ± 4.9 (27.4)	0.035	29.3 ± 6 (28.3)	30.28 ± 7.1 (28.2)	0.923
	<22	46 (27.5)	33 (40.7)	0.111	9 (22.5)	21 (32.8)	0.774
	22–27	14 (8.4)	6 (7.4)		5 (12.5)	5 (7.8)	
	>27	107 (64.1)	42 (51.9)		26 (65)	38 (59.4)	

Abbreviations: GCS: good cognitive status, CI: cognitive impairment. Values are presented as mean ± standard deviation (median). *p*-value represent statistical significance for T-test or Mann–Whitney for, respectively, normal or non-normally distributed data. Categorical variables are represented as total count (percentage). *p*-value represents statistical significance for Pearson Chi-square.

**Table 3:** Binary logistic regression models for low cognitive status vs. good cognitive status in the total sample (N = 352).

	Model 1	*p*-Value	Model 2	*p*-Value	Model 3	*p*-Value
Socio-demographic parameters						
Age ≥ cut-offs *	4.6 (2.48–8.54)	<0.001	3.32 (1.84–5.96)	<0.001	3.7 (2.1–6.55)	<0.001
Living in Beirut	4.6 (2.48–8.54)	<0.001	4.26 (2.18–8.3)	<0.001	4.51 (2.37–8.61)	<0.001
Anthropometric and health-related parameters						
Frailty			2.24 (1.12–4.49)	0.022		
Health perception			1.69 (1.01–2.82)	0.044	2.01 (1.24–3.26)	0.005
Dietary patterns						
MOD-MEDDP					1	0.033
WDP					2.04 (0.98–4.25)	0.058
HI-MEDDP					1.87 (1.07–3.29)	0.029

* Age cut-offs used were 80 years and 78 years for the literate and illiterate participants, respectively. Abbreviations: WDP: Westernized dietary pattern; HI-MEDDP: high-intake Mediterranean dietary pattern; MOD-MEDDP: moderate-intake Mediterranean dietary pattern.

**Table 4:** Binary logistic regression models for low cognitive status vs. good cognitive status in the literate sub-sample (N = 248).

	Model 1	*p*-Value	Model 2	*p*-Value	Model 3	*p*-Value
Socio-demographic parameters						
Age > 80 years	2.89 (1.46–5.76)	0.002	2.49 (1.14–5.42)	<0.001	2.24 (1.07–4.71)	0.033
Living in Beirut	3.73 (1.73–8.04)	0.001	2.25 (1.09–4.63)	0.027	5.21 (2.26–11.99)	<0.001
Anthropometric and health-related parameters						
Frailty			3.74 (1.49–9.42)	0.005	4.3 (1.66–11.14)	0.002
Dietary patterns						
MOD-MEDDP					1	0.018
WDP					3.08 (1.22–7.8)	0.017
HI-MEDDP					2.11 (1.05–4.22)	0.035

Abbreviations: WDP: Westernized dietary pattern; HI-MEDDP: high-intake Mediterranean dietary pattern; MOD-MEDDP: moderate-intake Mediterranean dietary pattern.

**Table 5:** Binary logistic regression models for low cognitive status vs. good cognitive status in the illiterate sub-sample (N = 104).

	Model 1	*p*-Value	Model 2	*p*-Value	Model 3	*p*-Value
Age > 78 years	5.84 (1.9–17.92)	0.002	5.39 (1.79–16.26)	0.003	5.08 (1.71–15.07)	0.003
Living in Beirut	6.59 (1.76–24.73)	0.005	5.4 (1.51–19.26)	0.009	5.85 (1.66–20.62)	0.006
Living conditions (compared with living alone)		0.007		0.025		0.019
Living with partner	6.12 (1.63–22.93)	0.007	4.04 (1.2–13.62)	0.025	4.13 (1.25–13.63)	0.020
Living with others	1.03 (0.3–3.52)	0.963	1.07 (0.31–3.67)	0.913	1.08 (0.33–3.61)	0.896

## Supplementary Materials

The following supporting information can be downloaded at: https://www.mdpi.com/article/10.3390/nu15183911/s1, Table S1: Characteristics of the total sample based on level of cognitive status; Table S2. Comparison between characteristics of literate and illiterate with low CS; Table S3: Health and functional status of participants living in Beirut and participants living in the other regions of Lebanon; Table S4. Nutritional & anthropometric characteristics: comparison between living in Beirut vs. living in other regions; Table S5. Comparison of nutrient intakes between participants living in Beirut vs. participants living in other regions; Table S6: Socio-demographic characteristics of participants living region in Beirut and participants living in the other regions of Lebanon; Table S7. Comparison of food consumption and dietary patterns based on cognitive status and level of literacy.
