# Supplementary material for: Effect of Mediterranean Dietary Pattern on Cognitive Status in Community-Dwelling Older Adults
Source: Nutrients. 2023 Sep 8;15(18):3911. doi: 10.3390/nu15183911 (PMC10537047; doi:10.3390/nu15183911)
Supplement: Supplementary file 1 [file nutrients-15-03911-s001.zip › nutrients-2573895-supplementary.pdf]

Supplementary Table S1. Characteristics of the total sample based on level of cognitive status.

N=352

N(%)		Good Cognitive status 207(58.8)	Low cognitive status 145(41.2)	P value
Age (years)		72 ± 7 (73)	75 ± 8 (74)	
Age80		25(12.1)	42(29)	<0.001
Income	Insufficient	110(53.1)	88(60.7)	0.16
	Sufficient	97(46.9)	57(39.3)	
Marital status	Married	136(65.7)	96(66.2)	0.92
Living conditions	Living with partner	118(57)	84(57.9)	0.482
	Living with others	61(29.5)	36(24.8)	
	Living alone	28(13.5)	25(17.2)	
Gender	Men	104(50.2)	72(49.7)	0.914
	Women	103(49.8)	73(50.3)	
Living in Beirut	Beirut	18(8.7)	43(29.7)	<0.001
BMI (kg/m^2)	<22	55(26.6)	54(37.2)	0.103
	22-27	19(9.2)	11(7.6)	
	>27	133(64.3)	80(55.2)	
		0.65 ± 0.08 (0.65)	0.65 ± 0.09 (0.64)	
WHTR	>0.718	38(18.9)	30(21.7)	0.522
Dependency	High	1(0.5)	9(6.2)	<0.001
	Low	20(9.7)	26(17.9)	
	No	186(89.9)	110(75.9)	
Physical Activity	Sedentary	165(79.7)	110(75.9)	0.629
	Regular active	40(19.3)	34(23.4)	
	Optimal active	2(1)	1(0.7)	
Multimorbidity		155(75.2)	115(79.3)	0.373
More than 2 age-related conditions		101(48.8)	88(60.7)	0.028
Polypharmacy		150(72.8)	104(75.4)	0.598
Frailty		19(9.2)	34(23.4)	<0.001
Malnutrition		5(2.4)	14(9.7)	0.003
Good health perception		102(50.2)	45(31.3)	<0.001
Dietary patterns	WDP	20(9.7)	22(15.2)	0.089
	HIMED-DP	43(20.8)	38(26.2)	
	MODMED-DP	144(69.6)	85(58.6)	

Values are presented as Mean ± standard deviation (median). P value represent statistical significance for T-test or Mann-Whitney for respectively normal or non-normally distributed data. Categorical variables are represented as N (percentage). P-value represent statistical significance for Pearson Chi-square.

Supplementary Table S2. Comparison between characteristics of literate and illiterate with low CS.

		Illiterate group	Literate group	p value
Age(yrs)		76.7±7.3 (76.5)	72.9±8.8 (72)	0.006
Living in Beirut	Beirut	24(37.5)	19(23.5)	0.066
	Other Region	40(62.5)	62(76.5)	
Gender	Men	24(37.5)	48(59.3)	0.009
	Women	40(62.5)	33(40.7)	
Income	Insufficient	45(70.3)	43(53.1)	0.035
	Sufficient	19(29.7)	38(46.9)	
Married	Married	40(62.5)	56(69.1)	0.402
Living conditions	Living with partner	36(56.3)	48(59.3)	0.680
	Living with others	15(23.4)	21(25.9)	
	Living alone	13(20.3)	12(14.8)	
BMI(kg/m2)		29.75±6.77 (28.14)	27.85±4.9 (27.35)	0.052
	<22	21(32.8)	33(40.7)	0.613
	22-27	5(7.8)	6(7.4)	
	>27	38(59.4)	42(51.9)	
WC(cm)		103.77±12.92 (101.7)	102.05±11.68 (101)	0.415
WHTR		0.68±0.1(0.67)	0.63±0.08(0.62)	0.002
	> 0.718	22(34.9)	8(10.7)	0.001
	<0.718	41(65.1)	67(89.3)	
HGS (kg)		18.35±7.64 (17.9)	26.38±9.12 (23.87)	0.001
	Low	49(77.8)	33(45.2)	<0.001
	Good	14(22.2)	40(54.8)	
Multimorbidity		56(87.5)	59(72.8)	0.030
Age-related conditions		49(76.6)	39(48.1)	0.001
Polypharmacy		42(70)	62(79.5)	0.200
Malnourishment		6(9.4)	8(9.9)	0.919
Good health perception		9(14.1)	36(45)	<0.001
Frailty		19(29.7)	15(18.5)	0.115
Mental health	Good	18(28.1)	36(45)	0.038
	Poor	46(71.9)	44(55)	
Physical Activity	Sedentary	52(81.3)	58(71.6)	0.164
	Regular active	11(17.2)	23(28.4)	
	Optimal active	1(1.6)	0(0)	
Caloric intake	Kcal/kg BW	26.4±8.9 (26.2)	28.3±9.2 (27.4)	0.225
Dietary patterns	WDP	9(14.1)	13(16)	0.947
	HIMED-DP	17(26.6)	21(25.9)	
	MODMED-DP	38(59.4)	47(58)	

Values are presented as Mean ± standard deviation (median). P value represent statistical significance for T-test or Mann-Whitney for respectively normal or non-normally distributed data. Categorical variables are represented as N (percentage). P-value represent statistical significance for Pearson Chi-square.

Supplementary Table S3. Health and functional status of participants living in Beirut and participants living in the other regions of Lebanon.

		Beirut (N= 61)	Other Regions (N=291)	P value
**Multi-morbidity**	**≤ 1 disease**	8 (13.3)	73 (25.1)	0.049
	**> 1 disease**	52 (86.7)	218 (74.9)	
**Age-related conditions**	**≤ 1 disability**	17 (27.9)	146 (50.2)	0.001
	**> 1 disability**	44 (72.1)	145 (49.8)	
**Polypharmacy**	**≥ 6 Medications**	43 (75.4)	211 (73.5)	0.763
	**< 6 Medications**	14 (24.6)	76 (26.5)	
**Physical Activity**	**Sedentary**	41 (67.2)	234 (80.4)	0.037
	**Regular active**	20 (32.8)	54 (18.6)	
	**Optimal active**	0 (0)	3 (1)	
**Dependency status**	**High dependency**	1 (1.6)	9 (3.1)	0.764
	**Low dependency**	9 (14.8)	37 (12.7)	
	**Independent**	51 (83.6)	245 (84.2)	
**Frailty status**	**Frail**	12 (19.7)	41 (14.1)	0.268
	**Not Frail**	49 (80.3)	250 (85.9)	
**Hand grip strength**	**Low HGS**	34 (56.7)	137 (50.2)	0.363
	**Good HGS**	26 (43.3)	136 (49.8)	
	**Strength (kg)**	23.5 ± 8.5 (22.7)	24.5 ± 9.6 (23.3)	0.585
**Fall during the past year**	**No falls**	41 (67.2)	216 (74.2)	0.456
	**Fall occurrence**	20 (32.8)	74 (25.4)	
**Cognitive decline**	**Good cognitive test**	34 (55.7)	202 (64.4)	0.039
	**Low cognitive test**	27 (44.3)	89 (30.6)	
	**Family History**	7 (11.5)	62 (21.3)	0.079
**Smoking status**	**Non-smoker**	24 (39.3)	142 (48.8)	0.388
	**Smoker**	24 (39.3)	93 (32)	
	**Previous smoker**	13 (21.3)	56 (19.2)	

Values are presented as Mean ± standard deviation (median). P value represent statistical significance for T-test or Mann-Whitney for respectively normal or non-normally distributed data. Categorical variables are represented as N (percentage). P-value represent statistical significance for Pearson Chi-square.

Supplementary Table S4. Nutritional & anthropometric characteristics: comparison between living in Beirut vs living in other regions.

		Beirut (N= 61)	Other Regions (N=291)	P value
**Nutritional status**	**poor**	37 (60.7)	90 (30.9)	<0.001
	**good**	24 (39.3)	201 (69.1)	
**BMI (kg/m^{2})**	**Mean**	29.8 ± 6.3	29 ± 5.7	0.221
	**BMI < 22**	21 (34.4)	88 (30.2)	0.724
	**BMI (22–27)**	4 (6.6)	26 (8.9)	
	**BMI > 27**	36 (59)	177 (60.8)	
**WTHR**		0.67 ± 0.11 [0.65]	0.64 ± 0.08 [0.64]	0.013

Abbreviations: BMI: body mass index; MAC: midarm circumference; WTHR: waist to height ratio; HSG: handgrip strength. Numeric variables are represented as median (interquartile range). Categorical variables are represented as N (percentage). Numeric variables are represented as mean ± SD [median]

Supplementary Table S5. Comparison of nutrient intakes between participants living in Beirut vs participants living in other regions.

	Beirut	Others	P value
	(N= 61)	(N=291)	
**Energy Cal/d**	1626.31 ± 398.41 [1627]	2000.42 ± 619.93 [1900]	0.001
**Protein (g/d)**	56.95 ± 16.34 [57.4]	70.61 ± 25.59 [68]	0.001
**Carbohydrates (g/d)**	180.54 ± 51.38 [177]	221.18 ± 77.22 [206]	0.006
**Sugars (g/d)**	56.65 ± 23.15 [53.4]	63.96 ± 30.26 [58]	0.556
**Fibers (g/d)**	16.68 ± 5.63 [16.5]	20.86 ± 7.8 [19.4]	0.003
**Fat (g/d)**	78.6 ± 20.37 [79.9]	97.28 ± 31.51 [92.5]	0.001
**SAFA (% TEI)**	12.9 ± 2.6 [12.2]	12.2 ± 2.72 [12.2]	0.346
**MUFA (% TEI)**	18.96 ± 3.88 [18.6]	19.71 ± 4.52 [19.1]	0.247
**PUFA (% TEI)**	8.63 ± 2.21 [8.5]	8.89 ± 2.53 [8.5]	0.792
**W3 FA (g/d)**	0.59 ± 0.3 [0.52]	0.89 ± 0.63 [0.69]	0.006
**W6 FA (g/d)**	11.09 ± 4.78 [10.6]	13.82 ± 6.56 [13]	0.12
**W6/W3 ratio**	19.95 ± 6.78 [20.76]	18.41 ± 7.87 [18.6]	0.355
**Cholesterol (mg/d)**	222.53 ± 127.42 [187]	252.44 ± 149.38 [223]	0.214
**VIT A (µg/d)**	765.98 ± 626.63 [543]	953.26 ± 839.86 [751]	0.582
**VIT D (µg/d)**	1.27 ± 0.83 [1.06]	1.34 ± 0.99 [1.1]	0.112
**VIT E (mg/d)**	9.47 ± 2.94 [9.02]	11.76 ± 4.19 [10.8]	0.02
**VIT K (µg/d)**	234.42 ± 163.62 [208]	317.09 ± 204.1 [272]	0.097
**VIT C (mg/d)**	89.73 ± 50.93 [78.5]	104.54 ± 57.01 [91.9]	0.284
**VIT B1 (mg/d)**	0.89 ± 0.26 [0.93]	1.12 ± 0.39 [1.05]	<0.001
**VIT B2 (mg/d)**	1.48 ± 0.49 [1.47]	1.69 ± 0.64 [1.64]	0.18
**VIT B3 (mg/d)**	17.36 ± 7.13 [15.9]	21.08 ± 8.92 [20.1]	0.041
**VIT B6 (mg/d)**	1.28 ± 0.38 [1.27]	3.15 ± 28.35 [1.49]	0.406
**VIT B9 (µg/d)**	293.15 ± 116.75 [278]	354.97 ± 136.37 [335]	0.047
**VIT B12 (µg/d)**	4.51 ± 4.35 [3.36]	5.54 ± 6.17 [3.86]	0.411
**Mg (mg/d)**	309.03 ± 115.5 [294]	371.73 ± 142.12 [355]	0.092
**Ca (mg/d)**	651.66 ± 212.95 [621]	765.58 ± 304.35 [733]	0.032
**P (mg/d)**	886.92 ± 239.91 [862]	1101.33 ± 386.14 [1069]	0.003
**Fe (mg/d)**	9.62 ± 2.9 [9.26]	12.37 ± 4.67 [11.6]	0.001
**Zn (mg/d)**	8.61 ± 2.47 [8.31]	10.38 ± 3.95 [10.3]	<0.001
**Se (µg/d)**	73.2 ± 28.54 [68]	88.92 ± 39.65 [86.8]	0.007

Supplementary Table S6: Socio-demographic characteristics of participants living region in Beirut and participants living in the other regions of Lebanon.

		Beirut (N= 61)	Other Regions (N=291)	P value
**Age (years)**		74 ± 7.1 [73]	73.1 ± 7.8 [73]	0.449
**Gender**	**Men**	29 (47.5)	147 (50.5)	0.673
	**Women**	32 (52.5)	144 (49.5)	
**Economic status**	**Insufficient**	43 (70.5)	155 (53.3)	0.014
	**Sufficient**	18 (29.5)	136 (46.7)	
**Marital status**	**Married**	36 (59)	196 (67.4)	0.217
	**Divorced**	4 (6.6)	6 (2.1)	
	**Single**	3 (4.9)	15 (5.2)	
	**Widowed**	18 (29.5)	74 (25.4)	
**Living conditions**	**Living with partner**	31 (50.8)	171 (58.8)	0.073
	**Living with others**	15 (24.6)	82 (28.2)	
	**Living alone**	15 (24.6)	38 (13.1)	
**Education**	**Illiteracy**	28 (45.9%)	79 (27.1%)	0.031
	**Less than elementary**	3 (4.9%)	39 (13.4%)	
	**Elementary**	15 (24.6%)	65 (22.3%)	
	**Complementary**	6 (9.8%)	40 (13.7%)	
	**Baccalaureate**	3 (4.9%)	37 (12.7%)	

Values are presented as Mean ± standard deviation (median). P value represent statistical significance for T-test or Mann-Whitney for respectively normal or non-normally distributed data. Categorical variables are represented as N (percentage). P-value represent statistical significance for Pearson Chi-square.

Supplementary Table S7. Comparison of food consumption and dietary patterns based on cognitive status and level of literacy.

	Literate			Illiterate		
	GCS	CI	*P value*	GCS	CI	*P value*
**Food & Food group**						
**Whole cereals & products**	1.8 ± 2.1 (1)	1.9 ± 2 (1.1)	0.628	1.4 ± 1.6(0.6)	1.1 ± 1.4(0.3)	0.326
**Refined flour products**	3.8 ± 3.3 (3.3)	3.4 ± 2.5 (2.9)	0.331	4.3 ± 2.6(4.3)	3.3 ± 2.5(3.4)	0.326
**Rice**	0.4 ± 0.4 (0.3)	0.5 ± 0.4 (0.4)	0.085	0.34 ± 0.32 (0.2)	0.4 ± 0.4 (0.3)	0.270
**Vegetables**	3.6 ± 1.6 (3.4)	3.7 ± 2 (3)	0.906	3.4 ± 1.9(3)	3 ± 2(2.4)	0.210
**Potato**	0.4 ± 0.3 (0.3)	0.3 ± 0.3 (0.3)	0.649	0.5 ± 0.5(0.3)	0.3 ± 0.3 (0.2)	0.042
**Fruits**	2.2 ± 1.3 (1.9)	2.4 ± 1.4 (2.4)	0.342	2.2 ± 1.3(1.9)	2.1 ± 1.2(1.9)	0.745
**Fruits & Vegetables**	5.9 ± 2.3 (5.6)	6.1 ± 2.7 (5.9)	0.546	5.6 ± 2.6 (5.5)	5 ± 2.6 (4.6)	0.263
**Milk & dairy products**	1.7 ± 1 (1.4)	1.6 ± 1 (1.6)	0.356	1.5 ± 0.8(1.3)	1.6 ± 0.8(1.5)	0.416
**Meat and poultry**	2.3 ± 1.5 (2.1)	2.3 ± 1.5 (1.9)	0.867	2.3 ± 1.3(2)	2.1 ± 1.2(1.9)	0.621
**Fish & shellfish**	0.6 ± 0.6 (0.4)	0.6 ± 0.6 (0.4)	0.604	0.5 ± 0.7(0.4)	0.6 ± 0.7(0.3)	0.822
**Eggs**	0.4 ± 0.5 (0.3)	0.4 ± 0.5 (0.3)	0.978	0.4 ± 0.3(0.3)	0.4 ± 0.4(0.3)	0.262
**Beans**	0.5 ± 0.5 (0.3)	0.6 ± 0.7 (0.5)	0.06	0.5 ± 0.4(0.6)	0.6 ± 0.8(0.3)	0.441
**Olives. seeds and oleaginous [illegible]**	5.8 ± 3.7 (4.9)	6.5 ± 4 (6.1)	0.192	5.6 ± 3.1(4.5)	6 ± 3.2(6)	0.486
**Vegetable oils**	2.6 ± 1.7 (3)	3 ± 1.8 (3)	0.085	2.8 ± 1.6(3)	2.7 ± 1.5(3)	0.552
**Processed & saturated fats**	0.4 ± 0.8 (0)	0.3 ± 0.6 (0)	0.212	0.4 ± 1(0)	0.2 ± 0.6(0)	0.352
**High Fat Sweets**	0.3 ± 0.5 (0.1)	0.4 ± 0.7 (0.1)	0.201	0.3 ± 0.6(0.1)	0.2 ± 0.3(0.1)	0.102
**Low Fat Sweets**	0.5 ± 0.8 (0.2)	0.9 ± 1.2 (0.3)	0.002	0.5 ± 0.9(0.2)	0.6 ± 1.1(0.2)	0.643
**Sugars and jams**	2.4 ± 3.2 (1.1)	3.3 ± 3 (2.5)	0.045	3.4 ± 4.9(1.7)	3.4 ± 4.4(2)	0.980
**Coffee**	3 ± 3 (3)	2.8 ± 2.4 (2)	0.841	3 ± 2.9 (2.5)	2.3 ± 1.9 (2)	0.155
**Tea**	0.8 ± 1 (0.3)	0.7 ± 0.9 (0.4)	0.786	1.1 ± 0.9 (1)	0.7 ± 0.9 (0.3)	0.029
**Dietary patterns**						
**WDP**	14(8.4)	13(16.05)		6(15)	9(14)	
**HI-MEDDP**	37(22.2)	21(25.9)	0.113	6(15)	17(26.6)	0.379
**MOD-MEDDP**	116(69.5)	47(58)		28(70)	38(59.4)	

Abbreviations: GCS: good cognitive status, CI: cognitive impairment.
Values are presented as Mean ± standard deviation (median).
P value represent statistical significance for T-test or Mann-Whitney for respectively normal or non-normally distributed data.
Categorical variables are represented as total count (percentage). P-value represent statistical significance for Pearson Chi-square.
